# The structure of the *Brassica napus* seed microbiome is cultivar-dependent and affects the interactions of symbionts and pathogens

**DOI:** 10.1186/s40168-017-0310-6

**Published:** 2017-09-01

**Authors:** Daria Rybakova, Riccardo Mancinelli, Mariann Wikström, Ann-Sofie Birch-Jensen, Joeke Postma, Ralf-Udo Ehlers, Simon Goertz, Gabriele Berg

**Affiliations:** 1 Graz University of Technology, Institute of Environmental Biotechnology, Petersgasse 12, 8010 Graz, Austria; 2 Department of Life Sciences, Università degli Studi di Trieste, Via L. Giorgeri, 34127 Trieste, Italy; 3 Agro Plantarum, Kärrarpsvägen 410, S-265 90 Åstorp, Sweden; 4 Wageningen Plant Research, 6708 PB Wageningen, Netherlands; 5 E-nema GmbH, Klausdorfer Str. 28-36, 24223 Schwentinental, Germany; 6 NPZ Innovation GmbH, Hohenlieth-Hof, 24363 Holtsee, Germany

**Keywords:** Plant microbiome, Seed microbiome, *Brassica Napus*, 16S rDNA amplicon sequencing, Confocal laser scanning microscopy, Cultivar specificity

## Abstract

**Background:**

Although the plant microbiome is crucial for plant health, little is known about the significance of the seed microbiome. Here, we studied indigenous bacterial communities associated with the seeds in different cultivars of oilseed rape and their interactions with symbiotic and pathogenic microorganisms.

**Results:**

We found a high bacterial diversity expressed by tight bacterial co-occurrence networks within the rape seed microbiome, as identified by llumina MiSeq amplicon sequencing. In total, 8362 operational taxonomic units (OTUs) of 40 bacterial phyla with a predominance of *Proteobacteria* (56%) were found. The three cultivars that were analyzed shared only one third of the OTUs. The shared core of OTUs consisted mainly of *Alphaproteobacteria* (33%). Each cultivar was characterized by having its own unique bacterial structure, diversity, and proportion of unique microorganisms (25%). The cultivar with the lowest bacterial abundance, diversity, and the highest predicted bacterial metabolic activity rate contained the highest abundance of potential pathogens within the seed. This data corresponded with the observation that seedlings belonging to this cultivar responded more strongly to the seed treatments with bacterial inoculants than other cultivars. Cultivars containing higher indigenous diversity were characterized as having a higher colonization resistance against beneficial and pathogenic microorganisms. Our results were confirmed by microscopic images of the seed microbiota.

**Conclusions:**

The structure of the seed microbiome is an important factor in the development of colonization resistance against pathogens. It also has a strong influence on the response of seedlings to biological seed treatments. These novel insights into seed microbiome structure will enable the development of next generation strategies combining both biocontrol and breeding approaches to address world agricultural challenges.

**Electronic supplementary material:**

The online version of this article (doi:10.1186/s40168-017-0310-6) contains supplementary material, which is available to authorized users.

## Background

The plant microbiome is a crucial factor for plant growth and health [1, 2]. The composition of plant microbiomes is remarkably robust relative to the complex and dynamic microbial environments in their surroundings [3]. A plant species-specific and even cultivar-specific component within the microbiome has been discovered using cultivation and microbial fingerprinting techniques [4, 5]. This was later confirmed by new sequencing technologies [6, 7]. Secondary metabolites of plants were identified as major driving forces in the regulation of microbial diversity and activity, while bulk soil was considered to be the main source of species richness [8]. Mosses, the phylogenetically oldest land plants on Earth, transfer a core microbiome containing plant beneficial bacteria via the sporophyte to the gametophyte [9]. The core microbiome of moss has been shown to be essential for germination [10]. In contrast to the assumption that the plant microbiome is predominantly assembled from external inoculum [11], seed-borne endophytes were recently found in pumpkin and maize [12, 13]. Moreover, seeds were found to be involved in the vertical transmission of microorganisms from one plant generation to the next [14]. Little is known, however, about the structure of seed-associated microbial assemblages and their impact on plant health.

*Brassica napus* L. (oilseed rape, canola) is an important oil-producing plant. Not only does rapeseed oil offer health benefits, it also shows potential as an alternative renewable energy source. The total area of world-wide cultivation of oilseed rape is increasing rapidly. In 2016, the FAO reported that over 71 million tons of rapeseed were being produced worldwide. Concurrently, substantial increases in yield losses caused by soil-borne pathogens have been reported. One such pathogen is *Verticillium longisporum* (C. Stark) Karapapa, Bainbr. and Heale (1997) [15]. This fungus was classified as a high risk plant pathogen affecting all *Brassicaceae. Verticillium* wilt infections are difficult to manage using conventional methods [16]. Plants lack resistance to most soil-borne pathogens, and they are consequently reliant on microbial-based defense mechanisms for their survival. These mechanisms are initiated and modulated by the plant through root exudates. Root exudates protect the roots by stimulating and enriching antagonistic microorganisms in the rhizosphere microbiome [17]. Nothing is known about the role of seed-associated microbiota in this respect. We hypothesize that the seed microbiota is a crucial factor for plant health in two ways: (i) diverse bacterial networks within seeds avoid pathogen transmission and (ii) tight bacterial networks lead to colonization resistance as suggested by Agler et al. [18] for the tight “hub” networks.

Microbial diversity in soil plays a key role against pathogens [19]. Intensive farming practices including narrow crop rotations, however, lead to a dysbiosis of the soil microbiome. The latter is associated with the increase of soil-borne pathogens and a decrease in plant-beneficial and symbiotic bacteria. Beneficial plant-associated microorganisms are a valuable resource for the development of biological control agents and plant growth promoting rhizobacteria [1, 5]. They can be applied in agriculture to improve plant health and to suppress pathogens [20]. Additionally, beneficial microorganisms support microbial diversity in the agricultural industry [21]. In order to develop biocontrol strategies that have consistently positive effects on plant health, we need to better understand the plant holobiont [2]. There has been very little published on the topic of the *Brassica* microbiome [22–25], and nothing is known about endophytic bacterial communities in *Brassica* seeds.

Three common cultivars, Avatar, Sherpa (NPZ), and Traviata (KWS SAAT AG), were chosen for our comparative study of the seed microbiota of *Brassica napus* L. A variety of methods were implemented in the experimental design in order to check our hypotheses. The structure of the bacterial communities associated with the seeds was studied using 16S rDNA amplicon sequencing. We were additionally able to visualize indigenous bacteria in seeds by using fluorescent in situ hybridization (FISH) combined with confocal laser scanning microscopy (CLSM). Insights into the function of seed microbiota were gained by PICRUSt and through interactive studies with symbionts and pathogens in the greenhouse and in the field.

## Methods

### Illumina MiSeq data processing and analysis

In order to study cultivar-dependency of the *B. napus* seed microbiome, analysis of the taxonomic composition of the bacterial communities was performed. Microbial DNA was extracted from the activated seeds of three commercially available oilseed rape cultivars Avatar, Sherpa, and Traviata. Twenty seeds per cultivar and replicate were washed three times each with sterile distilled water and were incubated for 4 h in 2 ml of water. Activated seeds were physically disrupted with sterile pestle and mortar and were resuspended in 2 ml of 0.85% NaCl under sterile conditions. The resultant suspensions were centrifuged (16,500×*g*, 20 min, 4 °C). The pellets were used for isolation of the total bacterial community DNA with the FastDNA Spin Kit for Soil and a FastPrep Instrument (MP Biomedicals, Illkirch, France) for 30 s at 5.0 ms^−1^. DNA was additionally purified by the GeneClean Turbo Kit (MP Biomedicals, Illkirch, France). The extracted DNA was treated with RNase (0.02 ng μl^−1^) for 5 min at 65 °C and was used as a template for a PCR. The 16S rRNA genes were amplified (three technical replicates for each sample) with the 515f and 806r primers [26]. The primers included sample-specific barcodes and Illumina cell flow adaptors. Peptide nucleic acid (PNA) PCR clamps were added to the PCR mix to block the amplification of plant host plastid and mitochondrial 16S DNA [27]. The PCR mixture (30 μl) contained 1 × Taq&Go (MP Biomedicals, Illkirch, France), 0.25 mM of each primer, 1.5 μM PNA mix, and 1 μl template DNA (94 °C for 3 min, 32 cycles of 94 °C for 45 s, 60 °C for 1 min, 72 °C for 18 s, and a final elongation at 72 °C for 10 min). Products were purified by the Wizard SV Gel and PCR Clean-Up System (Promega, Madison, WI, USA). DNA concentrations were measured using Nanodrop 2000 (Thermo Scientific, Wilmington, DE, USA). Equimolar aliquots of all samples were combined for amplicon sequencing using Illumina MiSeq v2 (250 bp paired end). Sequencing and raw sequencing data preparation, including joining forward and reverse read pairs, was performed by GATC Biotech AG (Konstanz, Germany). Data analysis was carried out using the software package QIIME 1.9.0 (http://qiime.org/). Sequence data was demultiplexed, and the reads were quality filtered by applying the phred quality threshold of 19. Reference-based chimera detection implemented in usearch 6.1 was used to detect chimeras, which were then removed. The remaining reads were clustered into operational taxonomic units (OTUs) at 97% similarity using a UCLUST algorithm with default parameters [28]. Taxonomic assignment of representative sequences was performed based on the reference database Greengenes release gg_13_8_99 [29]. Prior to further analysis, all reads assigned to plant plastids (chloroplasts and mitochondria) were discarded from datasets. The dataset was normalized to the lowest number of read counts (1670 reads per sample) for further analysis. Principal Coordinate Analysis (PCoA) was performed to assess the beta diversity based on the calculation of the weighted UniFrac distance matrix [30]. Ring-charts were created using the Krona software package version 2.7 [31]. The profile network on taxa level was constructed using Cytoscape version 3.4.0 [32]. Correlation and network analysis was performed on the data containing the core microbiome of all four replicates of each cultivar using CoNet extension of the Cytoscape, in accordance with the CoNet tutorial (http://psbweb05.psb.ugent.be/conet/microbialnetworks/conet.php). The parameter for significance was chosen based on the suggestion of Erlacher and co-workers [33]. Possible functions of the marker gene analysis were predicted with PICRUSt [34] according to the tutorial (http://picrust.github.io/picrust/index.html) and Galaxy modules provided by the Huttenhower lab. Statistical tests based on the OTU table for cases in which the sample means were unequal and the distributions were not normal were performed with the non-parametric ANOVA Kruskal-Wallis test, as suggested by Erlacher and co-workers [21]. The raw sequence files supporting the findings of this article are available in the NCBI Sequence Read Archive (SRA) under the BioProject ID PRJNA358488.

### Quantitative PCR (qPCR)

Microbial abundance in the seeds of oilseed rape cultivars Avatar, Sherpa, and Traviata, was determined by performing qPCR. Primers 515f—927r (10 μM each) were used to target the bacterial/archaeal 16S rRNA gene variable region 4 [26]. The qPCR reaction mix for bacteria contained 5 μl QuantiTect SYBR® Green PCR kit (QIAGEN GmbH, Hilden, Germany), 0.2 μl BSA, 0.12 μl forward and reverse primers, 0.8 μl PCR grade water, and 0.8 μl of the extracted genomic DNA. Amplification of DNA templates and quantification of fluorescence was achieved on a Rotor-Gene™ 6000 real-time rotary analyzer (Corbett Research, Sydney, Australia) as follows: 20 s at 95 °C, 15 s at 54 °C and 30 s at 72 °C for 40 cycles followed by a melt curve from 72 to 95 °C. Individual qPCR runs were performed in triplicate for each biological replicate. Occasional gene copy numbers found in negative controls were subtracted from their respective samples. The normalization by copy number was carried out in order to estimate the ratio of 16S copy numbers obtained by qPCR to the numbers of bacterial cells present within the seed. In order to normalize the 16S data, PICRUSt [34] was used guided by the tutorial (http://picrust.github.io/picrust/index.html) and Galaxy modules provided by the Huttenhower lab. The output of the normalization was used for the estimation of the mean 16S copy number for all assigned bacterial OTUs present in the oilseed rape seed microbiome (3.2 16S gene copies per cell). The estimated copy numbers were divided by a mean of bacterial 16S gene copy numbers per cell (3.2).

### Bacterial strains and growth conditions

The bacterial strains used in this study are listed in Table 1. Bacterial strains were routinely grown in Standard I nutrient agar (NA, SIFIN, Berlin, Germany) at 30 °C.

**Table 1:** Bacterial isolates used in this study

Strains	Closest database match	Environmental source	Reference
288P4R	*Burkholderia sordidicola*	*Lobaria pulmonaria*	Cernava et al. (2015)
Sb3-1	*Paenibacillus polymyxa*	Agricultural soil	Köberl et al. (2013)
CKB26	*Pseudomonas brassicacearum*	Brassica plants	This study
315P5BS	*Pseudomonas* sp.	*Lobaria pulmonaria*	Cernava et al. (2015)
HRO-C48	*Serratia plymuthica*	Oilseed rape rhizosphere	Müller et al. (2008)

### In vitro antagonistic assays

For selection of suitable strains for the *in planta* studies, the activity of the preselected bacterial strains towards *V. longisporum* was estimated. The preselected isolates (Table 1) were screened for their activity towards *V. longisporum* ELV25 Stark [15] (strain collection TU Graz, Environmental Biotechnology) by a dual culture in vitro assay on Waksman agar according to Berg et al. [35]. All strains were tested in three independent replicates.

### Plant growth in germination pouches

The cultivar-dependent effect of the selected bacterial strains on the growth of oilseed rape seedlings, as well as their respective seed colonization abilities were studied using gnotobiotic soil free assay. The winter oilseed rape *Brassica napus* L. partim cultivars Avatar and Sherpa (NPZ, Germany; Additional file 1: Figure S1) as well Traviata H 605886 (KWS Saat Einbeck, Germany) were bio-primed with the *V. longisporum* antagonistic bacterial strains *Pseudomonas brassicacearum* CKB26, *Burkholderia sordidicola* 288P4R and *Pseudomonas* spp. 315P5BS (Table 1) following the protocol described in Rybakova et al. [36]. The plant growth promoting capacity and the seed and root-colonization ability of each strain was estimated [36]. The weights of the green parts of the 14-day-old seedlings were compared to the untreated control seedlings of the same cultivar. The experiment was carried out in 4 replicates for each strain with 14 bio-primed seeds from each cultivar. Plant growth promoting effects of the microorganisms were statistically analyzed using the IBM SPSS program version 20.0 (IBM Corporation, Armonk, NY, USA). The significance of the differences in plants’ weights between the non-inoculated control versus each treatment group was calculated using a pairwise *t* test with independent samples. The decision to make use of the non-parametric Mann-Whitney *U* test as an alternative to the *t* test was based on assessment of the distributions of variables (normal versus non-normal). Data was expressed as the geometric mean ± standard deviation.

### Greenhouse trial and inoculation with *V. longisporum* ELV25

The cultivar-specific effect of the selected bacterial strains was additionally tested under greenhouse conditions using healthy and infested field soil. The seeds of three cultivars used in the greenhouse trials were treated with a beneficial *P. polymyxa* strain Sb3-1 (Table 1) using the bio-priming method as described above. This yielded concentrations of 0.29, 0.40, and 0.46 × 10^6^ CFU per seed (in Traviata, Sherpa, and Avatar, respectively). Plants were grown in three different soils: (1) disease-free field soil, (2) field soil infested with *V. longisporum* VL25, and (3) a field soil with a history of natural infection with *Verticillium* wilt. Swedish arable soils were collected from a disease free field and two fields which contained natural infection with *Verticillium* (Sireköpinge and Köpingsberg, 55° 55′ 28.9367″ N13° 0′ 19.646″ E, and 55° 55′ 21.3191″ N13° 0′ 54.8709″ E, respectively). Half of the disease-free soil was artificially infested with 10^3^ microsclerotia of *V. longisporum* ELV25 per ml as described below. Microsclerotia of *V. longisporum* ELV25 were produced in perlite with Czapek Dox following the protocol of Postma et al. [37]. Perlite (300 ml) and 500 ml of Czapek Dox solution (Sigma-Aldrich, Germany) were sterilized in a 1-l Erlenmeyer flask. The excess solution was then decanted. The medium was then inoculated with 30 punches of the fungus grown on potato dextrose agar and was left for 4 weeks at 20 °C in the dark with careful shaking after 3 weeks. The medium with microsclerotia was then dried and blended. The microsclerotia concentration was enumerated by microscope and was diluted in silver sand before being used. The microsclerotia were mixed with the sand-potting soil mixture in a concentration of 10^3^ microsclerotia/ml soil (equivalent to 1.3 10^6^ microsclerotia/pot). Pots were filled with 1.4 l of the soil. Each treatment consisted of 12 pots. Germination was assessed using two seeds per pot. Residual plants were removed after 22 days, leaving one remaining plant per pot. Disease development was followed for a period of 10 weeks after sowing. The greenhouse was maintained at 23/18 °C day/night with 16/8 h light/dark. Pots were watered regularly and were fertilized once per week with a nutrient solution. Statistical analysis of the greenhouse experiments were conducted with Genstat 17th Edition (Rothamsted Experimental Station, Harpenden, UK). After an analysis of variance, least significant difference (LSD) was calculated at a significance level of *p* = 0.05.

### Field trials

Over the course of the 2 years, two field trials were conducted with preselected bacterial strains, and two winter oilseed rape cultivars (Avatar and Sherpa). During the first year (2015–2016), the seeds of Avatar and Sherpa cultivars were treated with *P. polymyxa* Sb3-1 using a seed coating method, and with *S. plymuthica* HRO-C48 using encapsulation and bio-priming methods. Different treatments were used because the shelf life of certain BCAs was not sufficient after treatment in preliminary experiments (data not shown). The 2- and 3-day-old cultures (200 ml each) of *S. plymuthica* HRO-C48 were centrifuged at 10.000 rpm for 20 min. After centrifugation, the pellets were resuspended in 0.1 M MgSO_4_ × 7H_2_0.

Bio-priming involved treating the seeds of Avatar and Sherpa cultivars with bacterial suspension for 2 h under agitation followed by drying in a fluid bed dryer at 38 °C. Control treatment was suspended in 0.1 M MgSO_4_ × 7H_2_O only.

In the process of coating oilseed rape seeds with encapsulated *S. plymuthica* HRO-C48, cells were encapsulated in alginate. A solution of sodium alginate (Fluka, St. Louis, USA; 1000 mL of a 2.5% (*w*/*v*)) was prepared with autoclaved, deionized water and was filtrated through a filter membrane with a pore diameter of 45 μm. The cells of *S. plymuthica* HRO-C48 (log_10_ 11.7) were added, and alginate beads were produced by dropping the alginate solution with compressed air through a nozzle with a diameter of 200 μm into stirred 0.1 M CaCl_2_ solution. After the alginate beads were completely hardened, they were sieved out and washed with deionized and autoclaved water. The alginate beads were directly air-dried under laminar flow at room temperature and ground to a fine powder in a coffee grinder. The oilseed rape seeds were coated with encapsulated *S. plymuthica* HRO-C48. Encapsulated bacteria (25 g) were coated on the seeds. The seeds were treated with Tetramethylthiuramdisulfid (TMTD, Satec, Germany) and finally dried with talcum. The control seeds were treated with TMTD and then with talcum.

The coating of seeds was performed using a dry powder of *P. polymyxa* Sb3-1 (5 × 10^8^ CFU g^−1^), followed by treatment with TMTD. The coated seeds were dried with talcum.

The field experiments were carried out at the Kärrarp and Lockarp site of oilseed rape production, Sweden (56° 9′ 29.6359″ N12° 59′ 28.0549″ E and 55° 32′ 22.5117″ N13° 0′ 2.2931″ E, respectively). Plot size was 2.5 × 12 m in the winter oilseed rape trial in Kärrarp and 3 × 12 m in Lockarp. The trials had four replicates of each treatment in a randomized block design. In Kärrarp, inoculum of *V. longisporum* was poured into the rows at the same time as the seeds at the time of sowing. The field in Lockarp had been previously, naturally infected with the *Verticillium* wilt, and it was therefore decided not to artificially infest the field with a pathogen. The number of plants that initially germinated was counted after fully emerging. The plants were counted a second time in the spring in order to determine how many plants had died over the course of the winter. Symptoms of *Verticillium* wilt were assessed, and the proportion of infested plants was calculated.

### FISH-CLSM and LIVE/DEAD BacLight stain

The plant colonization patterns of *P. brassicacearum* CKB26 and *S. plymuthica* HRO-C48 were additionally studied using the in-tube FISH technique followed by visualization using CLSM [36, 38]. All FISH probes were purchased from genXpress GmbH (Wiener Neudorf, Austria). EUB338MIX (Cy3-labeled) was used for staining overall bacterial communities [39]. *Pseudomonas* spp. were visualized using the *Gammaproteobacteria* specific probe GAM42a labeled with Cy5 dye. The unlabeled *Betaproteobacteria* competitor probe (BET42a-competitor) was added to the GAM42a in equimolar proportion in order to minimize the unspecific bindings to *Betaproteobacteria* [40]. The *B. sordidicola* 288P4R was visualized using a *Betaproteobacteria*-specific probe BET42a labeled with ATTO488 mixed in equimolar proportion with an unlabeled *Gammaproteobacteria* competitor probe (GAM42a-competitor) [40]. The unspecific binding of the probes to the plants or bacteria was analyzed by including a negative control sample treated with NONEUB-FITC and NONEUB-Cy3 probes for the first and second hybridization steps, respectively. In order to intensify the resolution of plant structure images, the samples were stained with calcofluor white (CFW), which binds to β-1,3 and β-1,4 polysaccharides. The sections were incubated with 350 μl of 0.15% CFW staining solution for 20 to 30 min in the dark and were then rinsed with ice cold double-distilled water. The plant tissues (CFW stain and autofluorescence) were excited with a 405 nm laser beam and were detected at 425–490 nm. The FISH stained samples were further mounted with SlowFade Gold Antifadent (Molecular Probes, Eugene, OR, USA) and were stored overnight at 4 °C. Observations of the samples were performed with a Leica TCS SPE confocal laser scanning microscope (Leica Microsystems, Mannheim, Germany) equipped with solid state and UV lasers. Confocal stacks were acquired with Z-step of 0.4–0.5 μm and sequential activation of laser lines/detection windows. Maximum projections of 0.4–1 μm-depth optical slices were applied to visualize the root and seed sections (confocal stacks). In order to visualize the bacterial communities in the seeds, the surface-sterilized seeds of both untreated seeds and seeds bio-primed with either *P. brassicaceae* CKB26 or *S. plymuthica* HRO-C48 (Table 1) were used. The seeds were sliced into 100 μm slices using Cryotom (Leica CM 3000 cryostat, GMI, USA). Bacterial strains were either fixed directly on slides followed by FISH-CLSM as described above or stained with LIVE/DEAD BacLight stain (Invitrogen), following the instructions provided by the manufacturer. The following bacterial probes were used for the FISH-CLSM of the seeds: *Alphaproteobacteria* were labeled with Alexa488-labeled ALF968 probes. Cy3-labeled EUB338MIX was used for staining overall bacterial communities [39]. The FISH staining and visualization of the samples was carried out as described above.

## Results

### Structure of the bacterial *Brassica* seeds microbiota

**Fig. 1:**
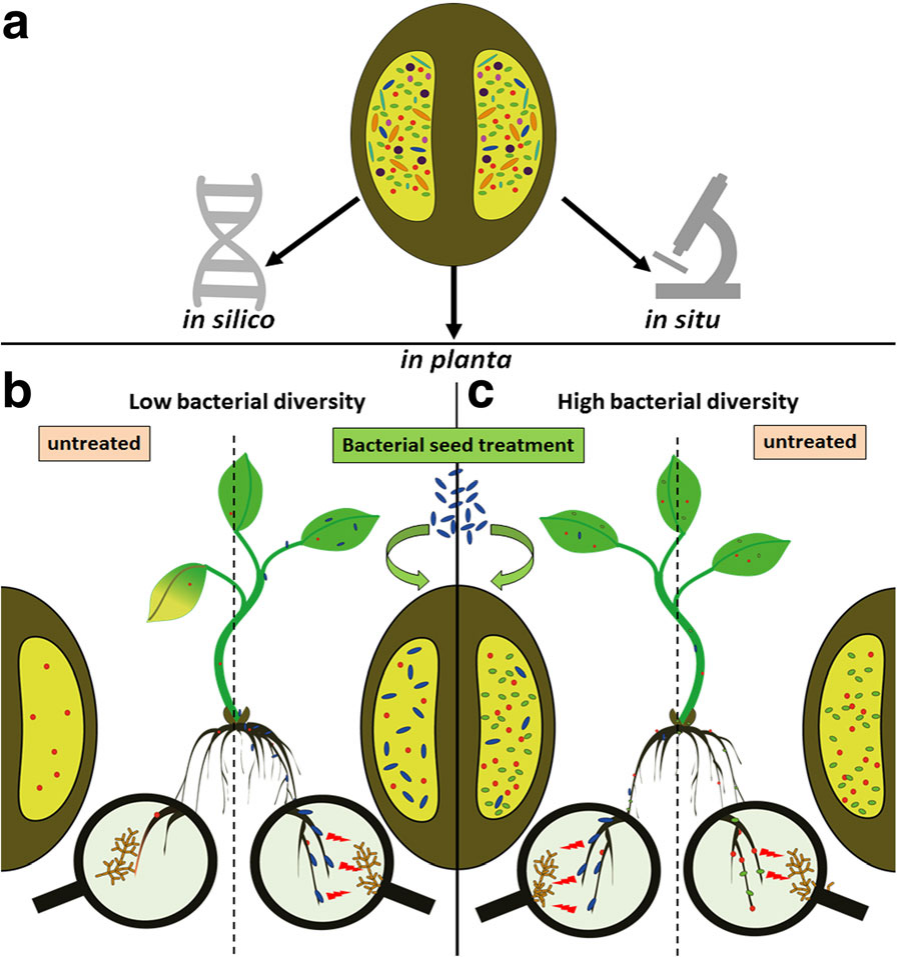
Structure of the study (**a**) and the suggested model linking bacterial diversity of the seed microbiota with colonization resistance against beneficial and pathogenic microorganisms (**b**, **c**). In our study, we combined three experimental approaches: a bioinformatic *in silico* approach, an experimental *in planta* approach, and a confocal microscopy *in situ* approach (**a**). The suggested model (**b**, **c**) explains the reactions of oilseed rape cultivars to the treatments with beneficial organisms. We compared cultivars whose seeds contain either high (Sherpa and Traviata) or low (Avatar) bacterial diversity. The seeds characterized by lower bacterial diversity and lower amount of beneficial taxa (schematically shown on the *left side* of the image) can be colonized by allochthonous cells (depicted as *blue rods*) in higher amounts than seeds with comparatively higher bacterial diversity (shown on the *right side*). Therefore, the treatment of seeds showing lower bacterial diversity with beneficial bacterial strains may result in increased resistance towards pathogens (depicted as *brown* fungal colonies). By contrast, under conditions in which seeds with higher bacterial diversity are treated with the same bacterial strains, the resistance of the seedlings to pathogens is less affected. This model is suggested for plant seeds with a tight bacterial network in which the introduction of new bacterial strains is rather challenging

**Fig. 2:**
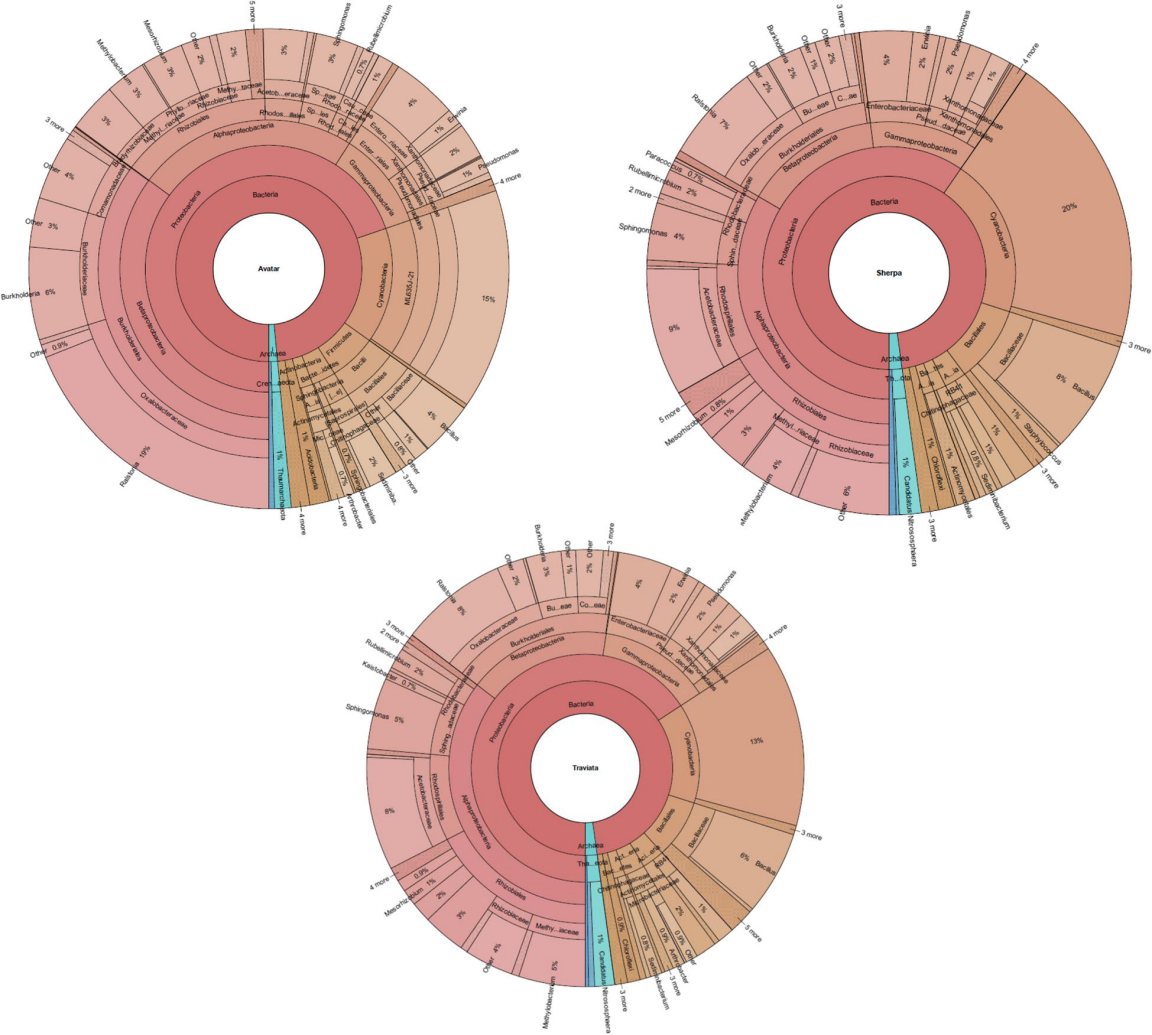
Ring-charts showing the bacterial community structures associated with the investigated oilseed rape seeds. The RDP classifier analysis is shown as derived from the mean of four samples per habitat. The *rings* represent different taxonomic rankings (order, family, and genus), and the *columns* represent distinct taxa. Minor taxa are not specified

We combined three experimental approaches in our study of the cultivar-dependent oilseed rape seed microbiome structure. These included sequencing, experimental, and microscopy (Fig. 1). In the first *in silico* approach, the bacterial diversity of the three commercially relevant cultivars of oilseed rape: Avatar, Sherpa, and Traviata were assessed by 16S rRNA gene amplicon sequencing. We obtained a total of 2,403,960 reads. After removing chimeras, control samples, mitochondrial, and chloroplast sequences, 9317 quality mean reads per sample (12 samples in total) remained (with a median absolute deviation of 4611.75 sequence reads). This corresponded with a total of 8362 OTUs. After normalization, the taxonomic assignment of OTUs revealed 40 bacterial phyla, 10 of which exceeded 1% of relative abundance (Additional file 1: Figure S2). The structure of the bacterial communities within the seeds of the three oilseed rape cultivars, including all taxonomic levels, is shown in the ring-charts in the Fig. 2. The seed microbiome of all three cultivars of oilseed rape contained mainly taxa of *Proteobacteria* (55.8%), followed by *Cyanobacteria* (12.7%) and *Firmicutes* (7.3%) (Additional file 1: Figure S2). *Alphaproteobacteria* was the most abundant class retrieved (26.4%), followed by *Betaproteobacteria* (17.8%) and *Gammaproteobacteria* (10.6%) (Additional file 1: Figure S2).

### Core and specific microbiome of *Brassica* seeds

**Fig. 3:**
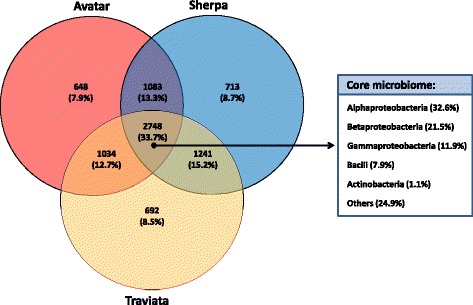
The taxonomic structure of the oilseed rape seed microbiota (*left*) and core seed microbiota (*right*). The taxonomic structure (*n* = 12) was retrieved at a 97% OTU cut-off level. The relative abundance of each phylum within the core microbiota is shown in *brackets* to the right. The *number* shown in brackets under each bacterial class name indicates its contribution to the overall core microbiota

From the 8362 of the total bacterial observed OTUs in all three cultivars, 2748 (33.7% of the total OTU) were shared between them (Fig. 3). Sherpa and Traviata shared the highest number of bacterial OTUs (1241 OTUs, which is 15.2% of the total OTUs). OTUs that were unique to each community represented 648 OTUs for Avatar (7.9% of total OTUs), 713 OTUs for Sherpa (8.7%), and 692 for Traviata (8.5%). This resulted in 25.1% cultivar specificity. The core microbiome of the *Brassica* seeds was further analyzed on the genus level and was sorted according to classes. We found 59 genera that represented 33.7% of all OTUs in the OTU table (Fig. 3). The core microbiome of oilseed rape seeds consisted of members of *Alphaproteobacteria* (32.6%), *Betaproteobacteria* (21.5%), *Gammaproteobacteria* (11.9%), *Bacilli* (7.9%), *Actinobacteria* (1.1%), as well as several other classes (24.9%) containing less than three different genera each (Fig. 3).

### Relative abundance, richness, and diversity analysis of bacterial communities

**Fig. 4:**
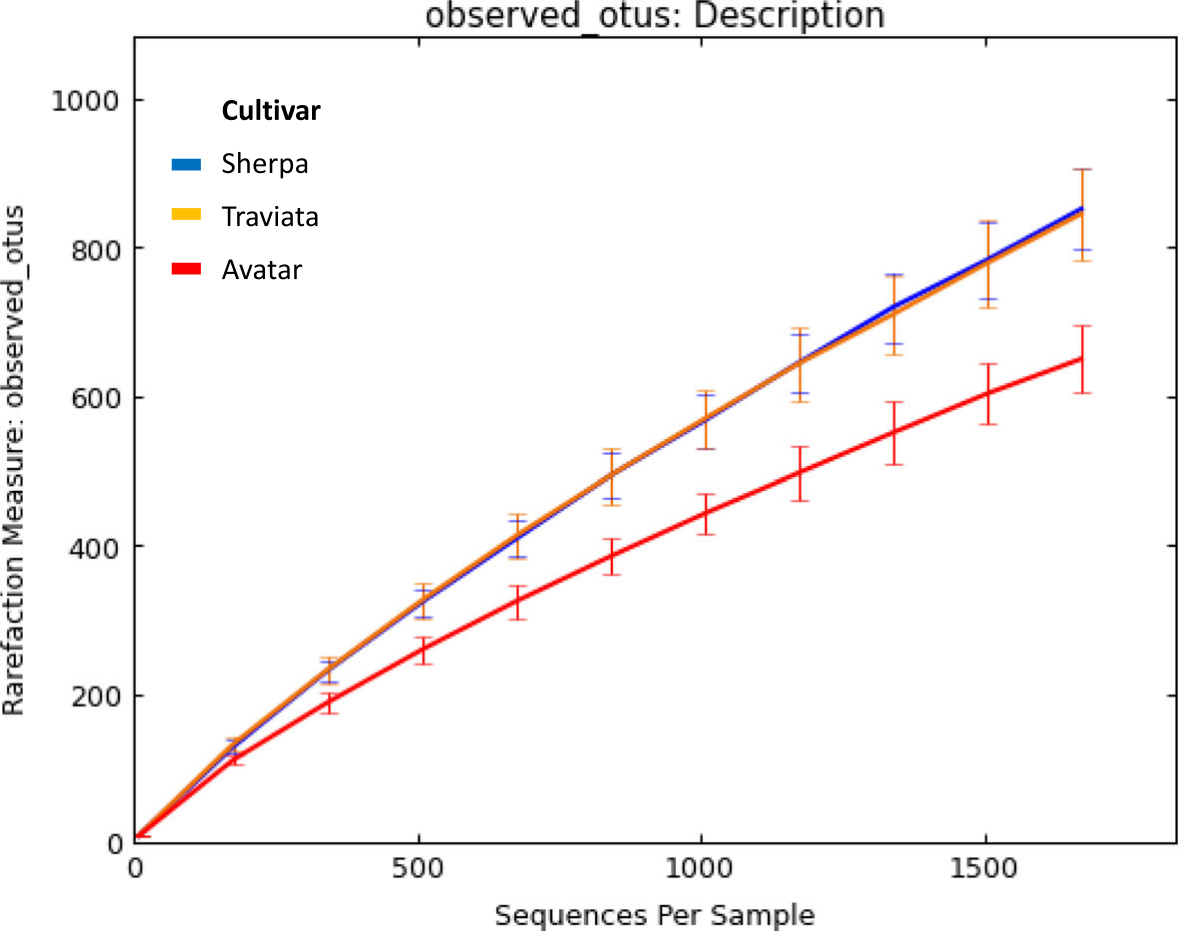
Overall diversity of the indigenous microbiota of the investigated oilseed rape seeds. The sample labeling was as follows: *red*: Avatar, *blue*: Sherpa, *orange*: Traviata oilseed rape cultivars

**Fig. 5:**
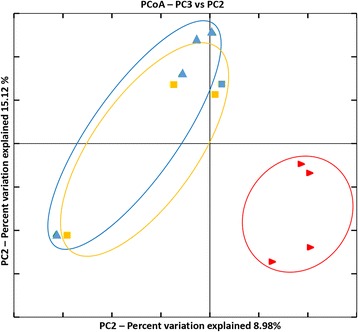
Beta diversity metrics of bacterial 16S rRNA genes among the seeds microbiomes of three investigated oilseed rape cultivars. Beta diversity community clustering is observed for phylogenetic beta diversity metrics (weighted UniFrac). In the panel, each point corresponds to a sample from either Avatar (*red triangles*), Traviata (*orange squares*) or Sherpa (*blue triangles*) seed samples. The percentage of variation explained by the plotted principal coordinates is indicated on the axes

The highest abundance of bacterial cells was found on the seeds of the Traviata cultivar (1.9 × 10^9^ ± 8.8 × 10^8^ bacterial cells per seed), followed by Sherpa (1.5 × 10^9^ ± 8.0 × 10^8^). Avatar demonstrated the lowest bacterial abundance among the cultivars (8.9 × 10^8^ ± 2.9 × 10^8^), however, these differences were not significant (Table 2). The alpha diversity analysis indices based on the observed species metric showed a significantly lower diversity of OTUs associated with the Avatar seeds (Fig. 4; Table 2). Beta diversity (pairwise sample dissimilarity) analysis based on weighted UniFrac distances was performed. It revealed distinctly clustered bacterial communities between the Avatar and the two other oilseed rape cultivars investigated. Sherpa and Traviata cultivars, on the other hand, clustered close together (Fig. 5).

**Table 2:** Overall abundancy and richness of the indigenous microbiota of the investigated oilseed rape seeds

Cultivar	Shannon^a^	Bacterial abundance^b^
Sherpa	9.1±0.3^a^	1.5 × 10^9^ ± 8.0﻿ × 10^8a^
Traviata	9.2 ± 0.1^a^	1.9 × 10^9^ ± 8.8 × 10^8^ ^a^
Avatar	8.3 ± 0.3^b^	8.9 × 10^8^ ± 2.9 × 10^8^ ^a^

Mean values and standard deviations (mean ± SD) are provided for seed samples. Values designated with the same letters were not significantly different (*p* < 0.05) according to a Tukey-HSD *t* test

^a^The richness was calculated using the Shannon estimator, and the graph was constructed using “observed OTUs” as the method of rarefication measurement

^b^The column labeled with (b) contains mean bacterial abundances per seed estimated using qPCR

### Relative abundances of putative beneficial/pathogenic microbiota in the cultivars and functional redundancy of PICRUSt predicted functions

Within the seeds belonging to the three cultivars, we compared the abundances of the major phyla commonly known to be beneficial for plants. Interestingly, 9 out of 11 beneficial phyla were found in lower abundance in Avatar seed microbiome when compared to Sherpa and Traviata seeds. Four of those phyla (*Acidobacteria, Chloroflexi, Planctomycetes,* and *Armatimonadetes*) were found to be significantly less abundant in the Avatar than in the two other cultivars (Additional file 1: Table S1). The taxa sorted on a genus level yielded a total of 747 distinct taxa, while 80 were statistically different among the cultivars (non-parametric ANOVA Kruskal-Wallis test, *p* ≤ 0.05). Of the 20 most abundant genera found in all seed cultivars, 9 are commonly recognized as being beneficial, and 3 genera (*Ralstonia, Salmonella*, and *Erwinia*) are considered to be potentially plant pathogenic (Table 3). We compared the 20 most abundant taxa with more than 1% relative abundance. Among those taxa, six were significantly increased, and two were significantly decreased in the Avatar cultivar when compared to the other cultivars (Table 3). Five of the genera found in higher abundance in the Avatar seeds were assigned to taxa that are recognized as being beneficial: *Burkholderiaceae, Bradyrhizobiaceae*, and *Mesorhizobium*. Plant beneficial *Pseudomonas*, on the other hand, was less abundant in the Avatar seeds compared to the other two cultivars*.* Notably, plant pathogenic *Ralstonia* was also significantly more abundant in Avatar. It yielded as much as 13.1% of the total seed microbiome, as compared to the 3.8% in Sherpa and 4.6% in Traviata (Table 3). We performed additional manual BLAST on the representative sequences with the most abundant OTUs assigned to the taxa *Ralstonia* and *Enterobacteriaceae* in order to verify the automatic assignment. In the case of the most abundant OTU originally assigned to *Enterobacteriaceae*, *Salmonella agona* had the highest identity score (523) followed by the *Escherichia coli* strain TYN 130606 (score 518). Both taxa showed 100% identity to the representative sequence, indicating that the assignment on the genus level was not possible. The manual BLAST of the most representative sequence of the abundant OTU belonging to the *Ralstonia* spp. showed the highest similarity rate with the two strains of *Ralstonia* (identity score of 518). The first match that appeared in a BLAST search was *Ralstonia insidiosa* ATCC 49129, a strain isolated from the sputum of a patient with acute lymphoblastic leukemia [41]. The second strain with an identical score belonged to *Ralstonia solanacearum*, a plant pathogenic species and quarantine organism.

**Table 3:** The most abundant taxa within investigated oilseed rape seed microbiomes

Taxa^a^	Avatar^b^	Sherpa^b^	Traviata^b^	Putative function^c^	Co-occurrence^d^
*Cyanobacteria* ML635J-21_	0.101 ± 0.026^a^	0.114 ± 0.073^a^	0.078 ± 0.041^a^	Beneficial	No
Ralstonia*	*0.131 ± 0.014* ^b^	0.038 ± 0.020^a^	0.046 ± 0.024^a^	Pathogen	Yes
*Acetobacteraceae*_	0.030 ± 0.007^a^	0.057 ± 0.025^a^	0.058 ± 0.021^a^	Beneficial	Yes
*Bacillus*	0.033 ± 0.015^a^	0.048 ± 0.012^a^	0.041 ± 0.008^a^	Beneficial	Yes
*Sphingomonas*	0.029 ± 0.004^a^	0.035 ± 0.004^ab^	0.044 ± 0.010^b^	Beneficial	Yes
Salmonella/ E. coli*	0.033 ± 0.008^a^	0.025 ± 0.005^a^	0.027 ± 0.008^a^	Pathogen	No
*Methylobacterium*	0.023 ± 0.004^a^	0.026 ± 0.007^a^	0.033 ± 0.010^a^	Methanotroph	No
*Burkholderia*	*0.044 ± 0.006* ^b^	0.015 ± 0.005^a^	0.016 ± 0.007^a^	Beneficial	Yes
*Methylocystaceae*_	0.019 ± 0.009^a^	0.021 ± 0.005^a^	0.026 ± 0.007^a^	Methanotroph	No
*Rhizobiaceae*	0.014 ± 0.001^a^	0.031 ± 0.022^a^	0.020 ± 0.006^a^	Beneficial	No
Acidobacteria_iii-1-15_	*0.013 ± 0.002* ^a^	0.026 ± 0.003^b^	0.025 ± 0.001^b^	Unknown	No
*Comamonadaceae*_	*0.032 ± 0.004* ^b^	0.011 ± 0.003^a^	0.015 ± 0.005^a^	Unknown	Yes
*Xanthomonadaceae*_	0.022 ± 0.002^a^	0.015 ± 0.002^a^	0.015 ± 0.004^a^	Beneficial and pathogen	Yes
*Bradyrhizobiaceae*_	*0.024 ± 0.004* ^b^	0.008 ± 0.003^a^	0.010 ± 0.003^a^	Beneficial	Yes
*Pseudomonas*	*0.010 ± 0.000* ^a^	0.014 ± 0.001^b^	0.016 ± 0.002^b^	Beneficial	No
*Rubellimicrobium*	0.009 ± 0.001^a^	0.015 ± 0.005^a^	0.015 ± 0.005^a^	Unknown	No
*Burkholderiaceae*_	*0.023 ± 0.003* ^b^	0.006 ± 0.003^a^	0.012 ± 0.007^a^	Beneficial	Yes
*Mesorhizobium*	*0.021 ± 0.002* ^b^	0.006 ± 0.001^a^	0.008 ± 0.001^a^	Beneficial	Yes
*Erwinia*	0.009 ± 0.000^a^	0.01 ± 0.003^ab^	0.014 ± 0.002^b^	Pathogen	No

^a^The OTU designation was performed at genus level where possible. The taxa for which no genus levels were assigned were named as the lowest known taxonomy followed by “_”. For the taxa labeled with *asterisks*, a manual BLAST was performed on the representative sequence of the most abundant OTUs. The assignment was based on the highest scores with at least 99% identity. In cases where several taxa with the same score appeared, both identifications were stated

^b^The relative abundance of the 20 most abundant (median among the cultivars and repetitions) taxa. The taxa that were significantly different (*p* < 0.05, according to a Tukey-HSD *t* test) in one of the cultivars when compared to the two others are highlighted in *italics*. Values designated with the same letters were not significantly different (*p* < 0.05) according to a Tukey-HSD t test

^c^The putative functions of some known strains of the taxa

^d^The involvement of the taxa in the statistically significant (*p* < 4 × 10^−4^; *q* < 4 × 10^−4^) co-occurrence patterns as shown by network analysis is labeled as “yes”, otherwise “no”. Please refer to the Fig. 5

The functional properties of taxa detected in 16S gene analysis of seed microbiome were predicted with PICRUSt. Most of the predicted bacterial functions were similar between the cultivars’ microbiomes (Additional file 1: Table S2), and indicated a high degree of functional redundancy. Several predicted functions of Avatar seed microbiota were found to be different from those of Sherpa and Traviata seed microbiota. For example, functions responsible for metabolism or degradation of several amino acids, benzoate, glyoxylate and dicarboxylate, aminobenzoate, glutathione, limonene, pinene, geraniol, chloroalkane, and chloroalkene were found to be altered in Avatar cultivar (Additional file 1: Table S3). The alpha diversity analysis showed no significant differences in the richness levels of predicted microbial functions associated with the seeds of the three cultivars tested (Additional file 1: Table S4). Notably, the Avatar cultivar displayed a non-significant decrease in the richness levels of predicted microbial functions.

### Microbial interaction networks in the *Brassica* seeds microbiome

**Fig. 6:**
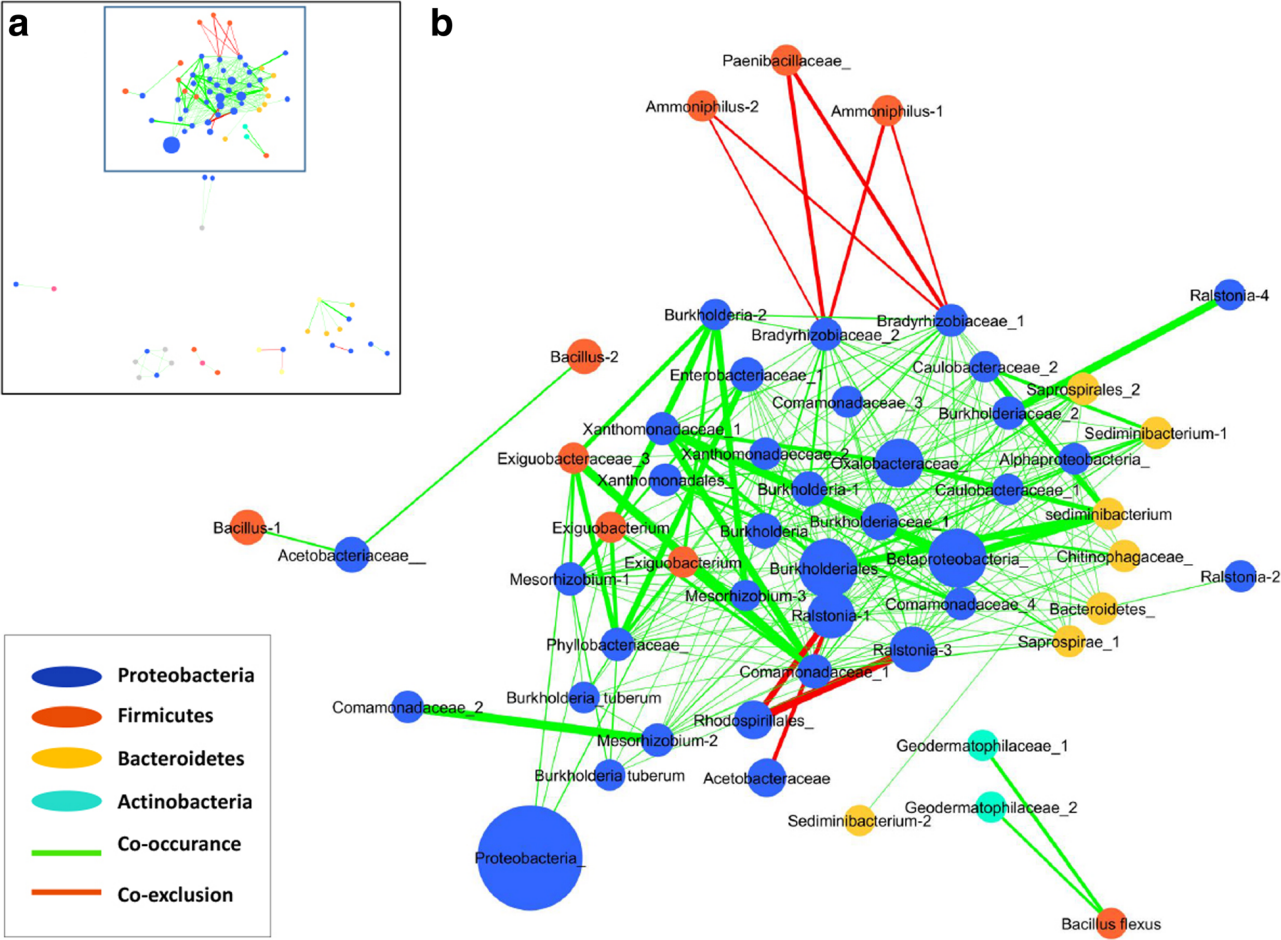
Significant co-occurrence and co-exclusion relationships among the oilseed rape seeds microbiome. Each node represents a bacterial order, describing one or more genus-level phylotypes. In cases where the identification of the OTU was not assigned on a genus level, higher-level taxonomic groups have been shown and labeled as “_” after the name. When several OTUs were assigned to the same taxa, the numbers (1–2) were added to the name in order to differentiate between the nodes. The color of nodes corresponds to the phylum (*blue Proteobacteria*, *red Firmicutes*, *orange Bacterioidetes*, *aquamarine Actinobacteria*), while the size of the nodes is proportional to their degree. Only significant interactions are shown (*p* < 4 × 10–4; q < 4 × 10–4). Edge width is proportional to the significance of supporting evidence, and *color* indicates the sign of the association (*red* negative, *green* positive). The image in **a** shows the full network highlighting the part of the network zoomed in the **b**

A microbial interaction network for the oilseed rape seed microbiome containing only significant interactions (*p* < 4 × 10^−4^; *q* < 4 × 10^−4^) and connected nodes is shown in Fig. 6. The network has a high complexity (80 nodes, network density 0.092, and average path length (2.019)), with a clustering coefficient of 0.210. The taxa involved in significant interactions are dominated by *Proteobacteria* (59.2%) followed by *Firmicutes* (14.1%), *Bacteroides* (12.7%), and *Actinobacteria* (4.2%). We found that the majority of the highly abundant taxa (as shown in Table 3), such as *Ralstonia, Acetobacteraceae, Bacillus, Sphingomonas, Burkholderiaceae, Comamonadaceae, Xanthomonadaceae, Bradyrhizobiaceae*, and *Mesorhizobium* demonstrated either strong co-occurrence or co-exclusion patterns (Fig. 6, Table 3). Among several positive interactions observed, we noted a strong co-occurrence relationship for some members of *Burkholderia* with *Exiguobacteraceae* and *Mesorhizobium*. One OTU belonging to the putative plant pathogen *Ralstonia* was positively correlated with the beneficial *Burkholderiaceae. Sediminibacterium* co-occurred with *Burkholderiales,* while *Comamonadaceae* correlated with *Mesorhizobium.* On the other hand, only a few co-exclusion relationships were observed among the significant interactions within the interaction network, as described below. We found that the occurrence of at least one OTU belonging to the plant pathogenic *Ralstonia* negatively correlated with the occurrence of N-fixating and P-mobilizing *Rhodospirillales* and plant beneficial *Acetobacteriaceae.* The occurrence of some OTUs belonging to the family of *Paenibacillaceae* (generally known for its high number of beneficial strains) and the genus *Ammoniphilus* (with an indeterminate function) correlated negatively with the occurrences of two different OTUs belonging to the putatively beneficial *Bradyrhizobiaceae* (Fig. 6).

### Cultivar-dependent response to the bacterial seed treatment under gnotobiotic conditions

Bacterial communities of the oilseed rape cultivars Traviata und Sherpa clustered close together in both the alpha- and beta-diversity microbiome analyses, while the Avatar cultivar microbiome showed significant differences (Figs. 4 and 5). We therefore decided to choose the Sherpa cultivar as the representative for both Sherpa and Traviata cultivars. We compared its response to the bacterial seed treatments with the Avatar cultivar’s response. In the process of selecting strains for all plant trials, we took into account both the combination of the in vitro activity of the strains and the results of the bioinformatics analysis. Preferred strains were those belonging to genera whose abundance was different among the cultivars, and those with the strongest in vitro activity against *V. longisporum*.

**Fig. 7:**
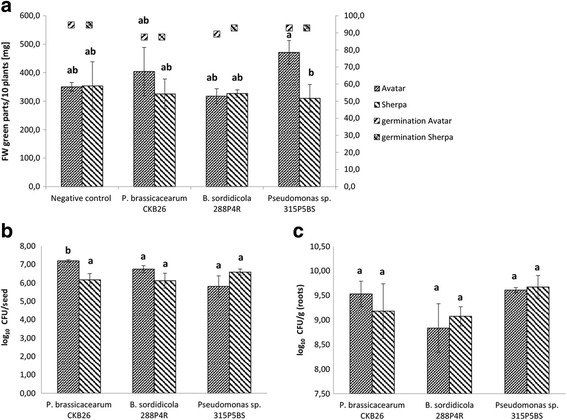
Effect of the three selected strains (*P. brassicacearum* CKB26, *Pseudomonas* sp. 315P5BS, and *B. sordidicola* 288P4R) on the 2-week-old seedlings of oilseed rape cultivars Avatar and Sherpa grown in germination pouches. The fresh weights of the green parts and seedlings’ germination are shown in **a**, while abundances of each strain on the seed (log_10_ CFU seed^−1^) and on the root (log_10_ CFU root^−1^) are presented in **b, c**, respectively. Values designated with the same letters were not significantly different (*p* < 0.05) according to a Duncan’s test

The surface-sterilized Sherpa and Avatar seeds were bio-primed with three plant-beneficial strains from genera *Pseudomonas* (*P. brassicacearum* CKB26 and *Pseudomonas* sp. 315P5BS) and *Burkholderia* (*B. sordidicola* 288P4R). These strains exhibited antagonistic activity towards *V. longisporum* Stark ELV25 in vitro. *P. brassicacearum* CKB26 was the significantly strongest antagonist of the fungal pathogen among tested strains (Table 4). In the case of both *Pseudomonas* treatments, Avatar seedlings grown in gnotobiotic soil-free conditions demonstrated stronger positive responses to bacterial treatment in comparison to the Sherpa seedlings (Fig. 7). We observed a significant difference in the plant growth promoting effects between Avatar and Sherpa seedlings treated with *Pseudomonas* sp. 315P5BS. The Avatar seedlings tended to produce more biomass than Sherpa seedlings when treated with *Pseudomonas* sp. 315P5BS. No significant differences were observed for both cultivars in comparison to the non-treated plants (Fig. 7a). The differences in the effects on the colonization of seeds and roots between both cultivars were insignificant with the exception of *P. brassicacearum* CKB26. The latter colonized Avatar seeds in significantly higher abundancies than Sherpa (Fig. 7b, c).

**Table 4:** Antagonistic activity of preselected *Pseudomonas* and *Burkholderia* strains towards *V. longisporum* Stark ELV25

Strains	The means of the zones of inhibition (mm)
*P. brassicaceae* CKB26	2.9 ± 1.0^a^
*Pseudomonas* sp. 315P5BS	1.0 ± 0^b^
*B. sordidicola* 288P4R	1.0 ± 0^b^

*The bacteria and *V. longisporum* were grown on Waksman agar. Zones of inhibition were measured and statistically analyzed after 4 days at 20 °C. According to the Tukey-HSD *t* test at *p* = 0.05, the means from three independent replicates that are followed by a common letter in each column and for each isolate do not differ significantly

### Cultivar-dependent response to the bacterial seed treatment in soil

The seeds of the Avatar, Sherpa, and Traviata winter oilseed rape cultivars were treated with *P. polymyxa* Sb3-1 and grown in three different soils under greenhouse conditions for 7 weeks. *P. polymyxa* Sb3-1 is a strain known for its antagonistic activity against *V. longisporum* [28]. We found no significant differences in the disease symptoms; however, we detected a potential trend in the way that the cultivars reacted to the seed treatment with the Sb3-1. Traviata oilseed rape cultivars reacted rather negatively to the bacterial treatment in the infested soil, resulting in a higher average amount of yellow leaves per plant. The same treatment showed positive effects on the Avatar cultivar in both artificially infested soil and in soil that was naturally infected with *Verticillium* (Table 5). We found no effect of the bacterial strains on the Sherpa cultivar under tested conditions.

**Table 5:** Antifungal effect of the *P. polymyxa* Sb3-1 on the 7-week-old seedlings of oilseed rape cultivars Traviata, Avatar, and Sherpa grown under greenhouse conditions in field soil

Soil	Treatment	number of yellow leaves per plant		
		Avatar	Sherpa	Traviata
Disease free	Sb3-1	0.9 ± 1.0	0.3 ± 0.8	0.6 ± 0.8
	Control	0.6 ± 0.8	0.5 ± 0.8	0.9 ± 1.1
Infested with *V. longisporum*	Sb3-1	1.3 ± 0.9	2.0 ± 1.2	2.6 ± 1.4
	Control	1.8 ± 1.3	2.2 ± 1.5	1.7 ± 1.2
Natural infection with *V. longisporum*	Sb3-1	0.9 ± 0.6	1.8 ± 0.8	1.4 ± 1.3
	Control	1.8 ± 1.2	1.9 ± 1.5	1.4 ± 1.3

**Fig. 8:**
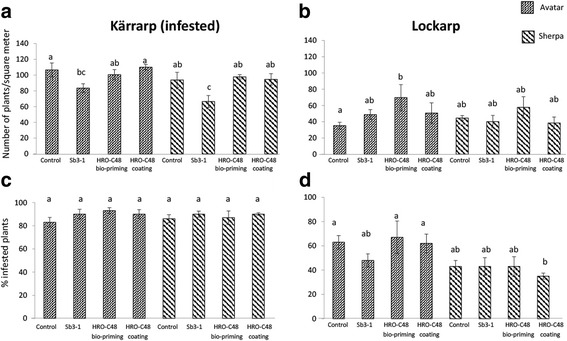
Effect of the selected strains (*P. polymyxa* Sb3-1, *S. plymuthica* HRO-C48 applied via bio-priming and *S. plymuthica* HRO-C48 applied via coating) on the germination rate and on the severity of *Verticillium* wilt infection of plants grown under field conditions. Oilseed rape cultivars Avatar and Sherpa were grown in Kärrarp (**a**, **c**) and Lockarp (**b**, **d**) locations in Sweden. Figure **a**, **b** shows the germination rate defined as “number of plants per square meter”, while Figure **c**, **d** illustrates disease rates defined as ratios of infested plants in relation to the total number of plants (%). Values designated with the same letters were not significantly different (*p* < 0.05) according to a Tukey-HSD *t* test

In order to reproduce the greenhouse results under field conditions, Avatar and Sherpa winter oilseed rape cultivars were treated with *P. polymyxa* Sb3-1 and *S. plymuthica* HRO-C48 using two different seed treatment methods. The latter was performed in order to determine whether varying seed treatment methods would have an impact on results. We observed a difference in the reaction of Avatar and Sherpa seedlings to treatment with both strains that was strongly dependent on the location (Fig. 8). The germination rate of the Avatar cultivar significantly improved following the bio-priming of the seeds with HRO-C48 in the Lockarp field, a field with a history of natural *Verticillium* wilt infection (Fig. 8b). This improvement in germination rate was not observed for the Sherpa cultivar when the same treatment was performed. In the field that was artificially infested with *Verticillium* (Kärrarp), the treatment of seeds with *P. polymyxa* Sb3-1 had a significantly negative impact on the germination of the seedlings of both cultivars (Fig. 8a). The same treatment resulted in a non-significant positive impact on the germination rate only in the case of the Avatar cultivar in the Lockarp field (Fig. 8b). During field trials, no significant differences in infection rates of *Verticillium* were detected relative to the untreated controls, however, the rate of plant infestation was less in the Avatar cultivar treated with *P. polymyxa* Sb3-1 (Fig. 8d). There were no significant effects of any of the seed treatments on the total yield (data not shown).

### Colonization patterns of the bacterial communities in oilseed rape seeds and seedlings

**Fig. 9:**
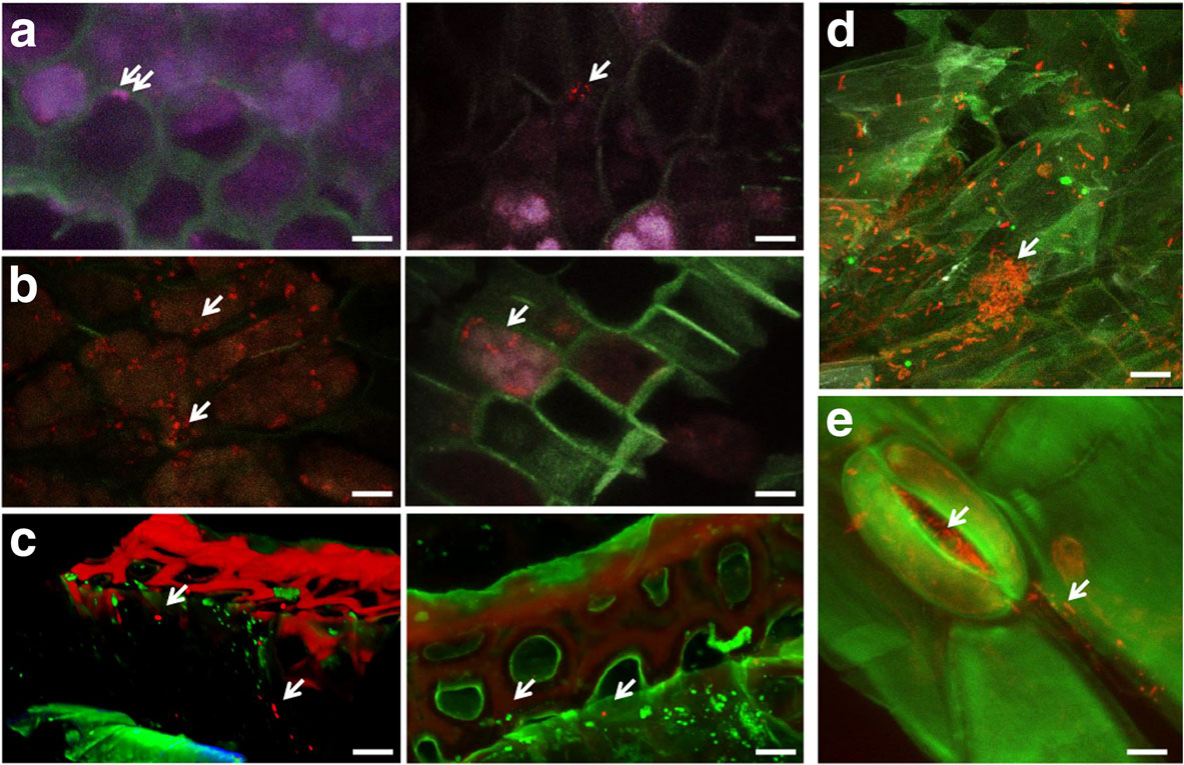
CLSM visualization of bacterial colonization patterns in the untreated (**a**) and bio-primed with *P. brassicacearum* CKB26 (**b**) and *S. plymuthica* HRO-C48 (**c**) oilseed rape seeds and in the rhizo- (**d**) and phyllosphere (**e**) of the oilseed rape seedlings. The strains in **a**, **b** were visualized using either FISH-CLSM using the *Alphaproteobacteria*-specific ALF968 probe labeled with Alexa488 (*blue*) and an equimolar ratio of eubacteria probe EUB338, EUB338II, and EUB338III labeled with the fluorescent dye Cy5 (*red*). In **c**, *Bac*Light LIVE/DEAD stain was used to visualize alive (*green*) and dead (*red*) *S. plymuthica* HRO-C48 in 3D projection. For the visualization of the *P. brassicacearum* CKB26 in (**d**) and (**e**), the *Gammaproteobacteria*-specific FISH probe GAM42a labeled with the fluorescent dye Cy5 (*green*) was overlaid with an equimolar ratio of eubacteria probe EUB338, EUB338II, and EUB338III labeled with the fluorescent dye Cy3 (*red*). Bacterial colonies are highlighted with *arrows*. *Bar* represents 10 μm

The colonization of the untreated surface-sterilized seeds as well as seeds bio-primed by *P. brassicacearum* CKB26 was visualized using CLSM. We were able to visualize bacterial cells in the unprimed seeds in a small quantity. They appeared mostly as individual cells or as small groups of two to five cells (Fig. 9a). The bacteria that were detected belonged either to the class of *Alphaproteobacteria* (Fig. 9a, left panel) or to other Eubacteria (Fig. 9a, right panel). Seeds treated with *P. brassicaceae* CKB26 (Fig. 9b) and *S. plymuthica* HRO-C48 (Fig. 9c) contained significantly higher amounts of bacterial cells than untreated seeds (Fig. 9a). The majority of the observed bacteria in the *S. plymuthica* HRO-C48 treated seeds were alive (Fig. 9c). Bacterial cells were found in both extracellular (Fig. 9b, left panel) and intracellular locations (Fig. 9b, right panel). Treatment of the oilseed rape seeds with the CKB26 strain resulted in the successful colonization of the rhizosphere and phyllosphere of the seedlings (Fig. 9d and e, respectively).

## Discussion

Our broader study of the *Brassica* seeds microbiomes revealed an unexpectedly high degree of cultivar specificity. By linking cultivar specificity with the reactions of cultivars to biocontrol treatments, we were able to confirm that seed microbiota is a crucial factor for plant health.

We identified high bacterial diversity as well as diverse bacterial networks within the seeds of all cultivars. The number of both live and dead bacterial cells present in each seed was estimated to be as much as two billion. The total number of OTUs identified among all samples was 8362 (measured at 97% identity). This amount was 34.5% higher than the amount of OTUs observed in the oilseed rape seed surface microbiome by Links et al. [24]. It was, however, 30% lower than the amount found in the microbiome of germinated *Brassica* seeds by Barett and co-workers [25]. The endophytic bacterial cells in the untreated seeds appeared either separately or in small groups as observed by FISH-CLSM. This suggests that endophytic cells cannot multiply and form colonies within the inactivated seeds before germination. The main phylum found in the seeds of all three cultivars was *Proteobacteria*. It was also the most common phylum observed in the oilseed rape root bacterial communities by De Campos et al. [23]. Barett et al. established *Gammaproteobacteria* as the main representative of *Brassica* seeds microbiota [25]. This class comprised more than 50% of the entire *Brassica* seed microbiome, however, our study showed a more evenly balanced distribution between *Proteobacteria* classes (24.6% *Alpha*-, 17.8% *Beta*-, and 10.6% *Gammaproteobacteria*). By analyzing bacterial networks, we found that co-occurrence, rather than co-exclusion, characterized the relationships among members of the root microbiota. We were able to establish that a great number of predominant taxa persisted and co-occurred with one another and with some minor taxa. A comparison of our study with other studies [6, 25, 42] allowed us to speculate that the microbial community structure of the oilseed rape seeds is especially tight. This may partially explain the challenges encountered in the development of biocontrol strategies for oilseed rape, as it is more difficult for the new strains to invade the tightly-knit bacterial community derived from the seed. Variations in seed production practices, storage conditions, and other abiotic factors may in part be responsible for variations in seed microbiota between seed charges, as suggested by Barett and co-workers [25]. However, plant species-specific and even cultivar-specific components in the microbiome structure have already been reported in many studies [5, 12, 13]. This confirms our hypothesis that the seed microbiome of oilseed rape is cultivar-specific. A high degree of cultivar specificity (25.1% cultivar-specific OTUs) was found in comparison to other studies [6]. Of the three cultivars studied, Avatar seeds contained the lowest amount of bacterial cells and showed a significantly smaller degree of overall bacterial diversity. Several potentially beneficial phyla and taxa were found in significantly lower abundance in the Avatar cultivar, while other potentially beneficial taxa occurred in lower abundance. We also found an unexpectedly high amount of potentially plant pathogenic *Ralstonia* spp. in the Avatar seeds (13.1%). Functional diversity within a microbiome has been postulated to be more important than structural diversity [2]. The PICRUSt prediction of the bacterial functions showed that several metabolic pathways were significantly different in Avatar seeds in comparison to the two other tested cultivars. This may indicate a higher rate of metabolic activity within the bacterial cells associated with this cultivar. Avatar is known to be more susceptible to the *Verticillium* wilt than the Sherpa cultivar (NPZ, personal communication), which is linked to the maturity level of the cultivar. The earlier the maturity level of the cultivar, the more it is prone to the development of the late wilt symptoms. Both hybrids show early seedling development, but differ in their root growth parameters. Avatar has a deep tap root and a high proportion of fine roots. Sherpa, on the other hand, generally has a larger root mass, and has a more pronounced lateral root system (NPZ, personal communication). The differences observed between cultivar root systems may be attributed to respective diversities of seed colonization by beneficial and pathogenic microorganisms. The seed microbiomes of cultivars may therefore have an impact on the growth of the plants and their respective levels of biotic and abiotic stress tolerance. Altogether, our observations of the structural as well as functional aspects of the oilseed rape seed microbiome confirmed our hypothesis that the seed microbiome of oilseed rape is cultivar-specific.

Further, we hypothesized that the generally low richness of the Avatar seed microbiome, high predicted metabolic activity, and reduction in the abundancies of the important beneficial phyla were factors contributing to its susceptibility towards pathogens. This hypothesis was verified by plant trials in which we compared different reactions of the cultivars to the bacterial treatments. Indeed, we found that Avatar was the only cultivar to show either a neutral or a mild positive antifungal effect when its seeds were treated with *P. polymyxa* Sb3-1. In one of the field locations (Lockarp), Avatar seedlings also reacted more strongly to the treatments with Sb3-1 and HRO-C48. In the second field (Kärrarp), we recorded a negative impact of the Sb3-1 treatment on the germination of both cultivars. A similarly negative impact of Sb3-1 on the oilseed rape seedlings was previously observed under the gnotobiotic soil-free conditions [36]. Avatar seedlings under gnotobiotic conditions also responded more strongly to the seed treatments with *Pseudomonas* spp. *P. brassicacearum* CKB26 was able to colonize seeds of the Avatar in significantly higher quantities than Traviata seeds. This outcome may be related to the significantly lower abundance of the *Pseudomonas* spp. in the Avatar seeds in comparison to abundances found in Sherpa and Traviata seeds. On the other hand, treatment with *B. sordidicola* 288P4R did not result in any measurable plant growth promoting effect. It is tempting to speculate that overall higher occurrence of *Burkholderia* spp. (0.025% in average for all three cultivars) is responsible for the absence of any effect of *B. sordidicola* 288P4R on the seedlings’ growth.

This is the first study that links the seed microbiome of commercially important oilseed rape cultivars with their ability to react to bacterial seed treatments. We proved a correlation between the diversity and tightness of the seed microbiome to the colonization resistance of the cultivars. Cultivars with a higher indigenous diversity were characterized by having a higher colonization resistance against beneficial and pathogenic microorganisms as illustrated in Fig. 1. The correlation of microbial diversity with colonization resistance towards pathogens was also demonstrated for the human gut microbiome [42, 43], thus confirming our theory of cross-kingdom similarity in host-microbial interactions [20].

## Conclusions

The seed microbiome of oilseed rape is cultivar-specific and reflects the impact of breeding. This was shown to influence the way in which cultivars interact with symbionts and pathogens. The structure of the seed microbiome determines the ability of plants to establish colonization resistance against pathogens and is therefore an interesting biomarker for breeding strategies. We recommend that seed microbiome studies be included in breeding strategies. Additionally, we believe that the assessment of seed microbiomes combined with network analysis may open new opportunities for targeted selection of biocontrol strains for a given host plant on the cultivar level. These novel insights into seed microbiome structure will enable the development of next generation strategies combining both biocontrol and breeding approaches to address world agricultural challenges.

## Additional file

Additional file 1: Table S1. Relative abundances of bacterial phyla in which plant-beneficial strains are currently recognized in Avatar, Sherpa, and Traviata oilseed rape cultivars. **Table S2.** Relative proportions of PICRUSt predicted functions of 16S rRNA marker gene with over 1% abundance in all samples. **Table S3.** Relative proportions of PICRUSt predicted functions of 16S rRNA marker gene with significant difference between the samples and over 0.3% abundance. **Table S4.** Alpha rarification analysis of predicted microbial functions*. **Figure S1.** Production sites of the Avatar and Sherpa seeds. **Figure S2.** Overlap of the different bacterial communities from oilseed rape seeds based on total OTU count (97% similarity). The OTU was regarded as present in a given cultivar if it was observed in at least one of the four replicates; the data was not normalized in order to include all observed OTUs. (DOCX 242 kb)

## Abbreviations

BSA: Bovine serum albumin
CFW: Calcofluor white
CLSM: Confocal laser scanning microscopy
DNA: *Deoxyribonucleic acid*
FISH: Fluorescent in situ hybridization
OTUs: Operational taxonomic units
PCoA: Principal Coordinate Analysis
PCR: Polymerase chain reaction
PICRUSt: Phylogenetic Investigation of Communities by Reconstruction of Unobserved States
qPCR: Real-time (quantitative) polymerase chain reaction
RNase: *Ribonuclease*
TMTD: Tetramethylthiuramdisulfid
