# Supplementary material for: The structure of the Brassica napus seed microbiome is cultivar-dependent and affects the interactions of symbionts and pathogens
Source: Microbiome. 2017 Sep 1;5:104. doi: 10.1186/s40168-017-0310-6 (PMC5580328; doi:10.1186/s40168-017-0310-6)
Supplement: Additional file 1: Table S1. — Relative abundances of bacterial phyla in which plant-beneficial strains are currently recognized in Avatar, Sherpa, and Traviata oilseed rape cultivars. Table S2. Relative proportions of PICRUSt predicted functions of 16S rRNA marker gene with over 1% abundance in all samples. Table S3. Relative proportions of PICRUSt predicted functions of 16S rRNA marker gene with significant difference between the samples and over 0.3% abundance. Table S4. Alpha rarification analysis of predicted microbial functions*. Figure S1. Production sites of the Avatar and Sherpa seeds. Figure S2. Overlap of the different bacterial communities from oilseed rape seeds based on total OTU count (97% similarity). The OTU was regarded as present in a given cultivar if it was observed in at least one of the four replicates; the data was not normalized in order to include all observed OTUs. (DOCX 242 kb) [file 40168_2017_310_MOESM1_ESM.docx]

**Supplemental**

**Table S1.** Relative abundances of bacterial phyla in which plant-beneficial strains are currently recognized in Avatar, Sherpa and Traviata oilseed rape cultivars

| **Phylum** | **Avatar^1)^** | **Sherpa^1)^** | **Traviata^1)^** |
| --- | --- | --- | --- |
| Cyanobacteria | 0.107±0.025^a^ | 0.124±0.067^a^ | 0.087±0.041^a^ |
| Firmicutes | 0.070±0.017^a^ | 0.083±0.011^a^ | 0.075±0.010^a^ |
| Acidobacteria | 0.032±0.003^a^ ↓ | 0.061±0.005^b^ | 0.060±0.005^b^ |
| Actinobacteria | 0.041±0.004^a^ | 0.049±0.004^ab^ | 0.055±0.006^b^ |
| Chloroflexi | 0.024±0.005^a^ ↓ | 0.044±0.003^b^ | 0.043±0.008^b^ |
| Bacteroidetes | 0.041±0.007^a^ | 0.036±0.004^a^ | 0.034±0.002^a^ |
| Planctomycetes | 0.013±0.002^a^ ↓ | 0.027±0.003^b^ | 0.022±0.001^b^ |
| Verrucomicrobia | 0.008±0.002^a^ | 0.016±0.002^b^ | 0.012±0.003^ab^ |
| Gemmatimonadetes | 0.008±0.001^a^ | 0.013±0.002^ab^ | 0.014±0.003^b^ |
| Nitrospirae | 0.003±0.001^a^ | 0.005±0.001^b^ | 0.004±0.000^ab^ |
| Armatimonadetes | 0.001±0.000^a^ ↓ | 0.003±0.001^b^ | 0.003±0.001^b^ |

**^1)^** Mean values and standard deviations (mean ± SD) are provided in oilseed rape seeds samples. Values designated with the same letters were not significantly different (P<0.05) according to a Tukey-HSD T-test.

**Table S2 Relative proportions of PICRUSt predicted functions of 16S rRNA marker gene with over 1% abundance in all samples**

| **Predicted function*** | **Avatar** | **Sherpa** | **Traviata** |
| --- | --- | --- | --- |
| Transporters | 5.92±0.12 ^a^ | 5.60±0.15 ^a^ | 5.75±0.18 ^a^ |
| ABC transporters | 3.68±0.08 ^a^ | 3.45±0.09 ^a^ | 3.54±0.10 ^a^ |
| DNA repair and recombination proteins | 2.17±0.03 ^a^ | 2.28±0.05 ^a^ | 2.24±0.02 ^a^ |
| Two-component system | 2.13±0.04 ^a^ | 2.10±0.03 ^a^ | 2.09±0.02 ^a^ |
| Purine metabolism | 1.79±0.02 ^a^ | 1.89±0.02 ^a^ | 1.87±0.02 ^a^ |
| Bacterial motility proteins | 1.84±0.01 ^a^ | 1.81±0.05 ^a^ | 1.72±0.06 ^a^ |
| Ribosome | 1.59±0.05 ^a^ | 1.74±0.07 ^a^ | 1.70±0.05 ^a^ |
| Oxidative phosphorylation | 1.41±0.02 ^a^ | 1.49±0.02 ^a^ | 1.45±0.04 ^a^ |
| Peptidases | 1.39±0.02 ^a^ | 1.48±0.02 ^a^ | 1.46±0.02 ^a^ |
| Transcription factors | 1.40±0.04 ^a^ | 1.35±0.05 ^a^ | 1.40±0.06 ^a^ |
| Pyrimidine metabolism | 1.20±0.02 ^a^ | 1.29±0.03 ^a^ | 1.27±0.02 ^a^ |
| Arginine and proline metabolism | 1.20±0.01 ^a^ | 1.23±0.02 ^a^ | 1.24±0.01 ^a^ |
| Amino acid related enzymes | 1.16±0.02 ^a^ | 1.22±0.01 ^a^ | 1.21±0.02 ^a^ |
| **Butanoate metabolism** | **1.19±0.02** ^b^↑ | 1.08±0.03 ^a^ | 1.11±0.01 ^ab^ |
| Pyruvate metabolism | 1.13±0.01 ^a^ | 1.12±0.01 ^a^ | 1.12±0.01 ^a^ |
| Carbon fixation pathways in prokaryotes | 1.03±0.02 ^a^ | 1.09±0.01 ^a^ | 1.07±0.02 ^a^ |
| **Propanoate metabolism** | **1.09±0.02** ^a^↑ | 0.98±0.02 ^a^ | 1.01±0.01 ^ab^ |
| Ribosome Biogenesis | 0.99±0.02 ^a^ | 1.05±0.03 ^a^ | 1.02±0.01 ^a^ |
| Glycolysis / Gluconeogenesis | 0.99±0.01 ^a^ | 1.02±0.00 ^a^ | 1.03±0.01 ^a^ |

* rows where a predicted function was found in a significantly higher abundance in Avatar seeds compared to at least one of the other cultivar were highlighted in **bold**. Mean values and standard deviations (mean ± SD) are provided in oilseed rape seeds samples. Values designated with the same letters were not significantly different (P<0.05) according to a Tukey-HSD T-test.

**Table S3 Relative proportions of PICRUSt predicted functions of 16S rRNA marker gene with significant difference between the samples and over 0.3% abundance**

| **Predicted function*** | **Avatar** | **Sherpa** | **Traviata** |
| --- | --- | --- | --- |
| Butanoate metabolism | **1.19**±0.02 ^b^↑ | 1.08±0.03 ^a^ | 1.11±0.01 ^ab^ |
| Propanoate metabolism | **1.09**±0.02 ^b^↑ | 0.98±0.02 ^a^ | 1.01±0.01 ^ab^ |
| Valine, leucine and isoleucine degradation | **1.03**±0.02 ^b^↑ | 0.92±0.04 ^a^ | 0.94±0.02 ^ab^ |
| Fatty acid metabolism | **0.91**±0.02 ^b^↑ | 0.78±0.02 ^a^ | 0.80±0.01 ^ab^ |
| Glyoxylate and dicarboxylate metabolism | **0.86**±0.01 ^b^↑ | 0.78±0.03 ^a^ | 0.80±0.01 ^ab^ |
| Benzoate degradation | **0.78**±0.03 ^b^↑ | 0.61±0.02 ^a^ | 0.64±0.03 ^ab^ |
| Tryptophan metabolism | **0.72**±0.02 ^b^↑ | 0.63±0.02 ^a^ | 0.66±0.01 ^ab^ |
| Lysine biosynthesis | 0.58±0.01 ^b^ | **0.61**±0.01 ^a^↑ | 0.60±0.01 ^ab^ |
| beta-Alanine metabolism | **0.59**±0.01 ^b^↑ | 0.50±0.01 ^a^ | 0.51±0.01 ^ab^ |
| Aminobenzoate degradation | **0.57**±0.02 ^b^↑ | 0.47±0.02 ^a^ | 0.49±0.01 ^ab^ |
| Tyrosine metabolism | **0.51**±0.01 ^b^↑ | 0.47±0.01 ^a^ | 0.48±0.00 ^ab^ |
| Glutathione metabolism | **0.50**±0.01 ^b^↑ | 0.47±0.01 ^a^ | 0.47±0.01 ^ab^ |
| Limonene and pinene degradation | **0.50**±0.02 ^b^↑ | 0.41±0.01 ^a^ | 0.42±0.01 ^ab^ |
| Phenylalanine metabolism | **0.44**±0.01 ^b^↑ | 0.40±0.03 ^a^ | 0.42±0.01 ^ab^ |
| Geraniol degradation | **0.47**±0.02 ^b^↑ | 0.37±0.01 ^a^ | 0.39±0.01 ^ab^ |
| Chloroalkane and chloroalkene degradation | **0.34**±0.01 ^b^↑ | 0.30±0.01 ^a^ | 0.31±0.01 ^ab^ |

***** the highest abundances among the seed samples for a predicted function were highlighted in **bold.** Mean values and standard deviations (mean ± SD) are provided in oilseed rape seeds samples. Values designated with the same letters were not significantly different (P<0.05) according to a Tukey-HSD T-test.

**Table S4 Alpha rarification analysis of predicted microbial functions***

|  | **Chao1** | **Observed OTUs** | **Shannon** |
| --- | --- | --- | --- |
| **Avatar** | **284.4±1.4 ^a^** | **282.8±1.8 ^a^** | **6.9±01 ^a^** |
| **Sherpa** | **286.94±3.2 ^a^** | **282.0±0.7 ^ab^** | **6.9±0.0 ^a^** |
| **Traviata** | **326.8±58.9 ^a^** | **288.0±4.1 ^b^** | **6.9±0.0 ^a^** |

The richness was calculated using the Chao1, observed OTUs and Shannon estimators. Mean values and standard deviations (mean ± SD) are provided for seed samples. Values designated with the same letters were not significantly different (P<0.05) according to a Tukey-HSD T-test.

**Fig. S1 Production sites of the Avatar and Sherpa seeds**

**
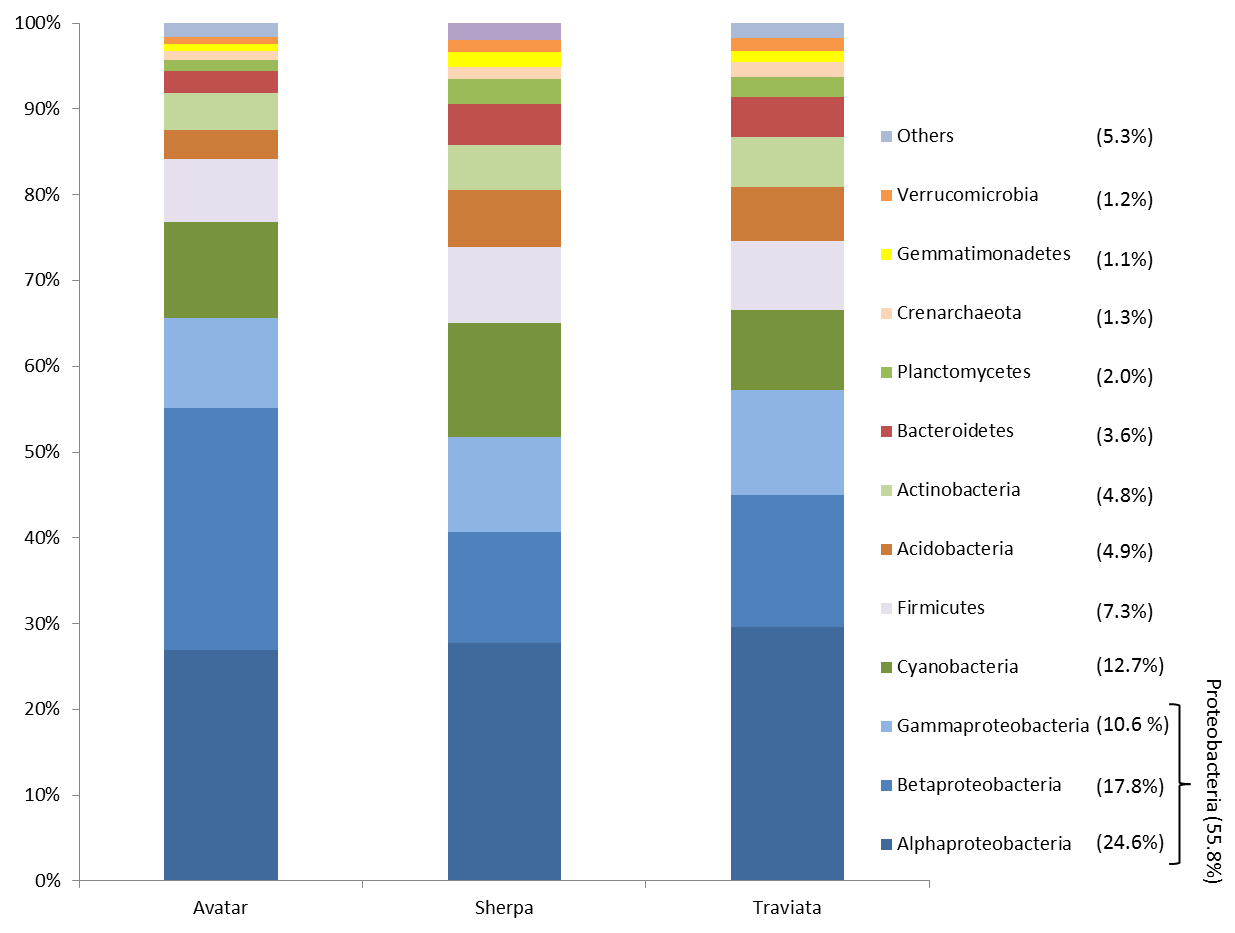
**

**Fig. S2. Overlap of the different bacterial communities from oilseed rape seeds** **based on total OTU count** (97% similarity). The OTU was regarded as present in a given cultivar if it was observed in at least one of the four replicates; the data was not normalized in order to include all observed OTUs.
